# Development of innovative multi-epitope mRNA vaccine against *Pseudomonas aeruginosa* using *in silico* approaches

**DOI:** 10.1093/bib/bbad502

**Published:** 2024-01-06

**Authors:** Maryam Asadinezhad, Saeed Khoshnood, Parisa Asadollahi, Sobhan Ghafourian, Nourkhoda Sadeghifard, Iraj Pakzad, Yasaman Zeinivand, Nazanin Omidi, Ali Hematian, Behrooz Sadeghi Kalani

**Affiliations:** Students Research Committee, Ilam University of Medical Sciences, Ilam, Iran; Department of Microbiology, Faculty of Medicine, Ilam University of Medical Sciences, Ilam, Iran; Students Research Committee, Ilam University of Medical Sciences, Ilam, Iran; Clinical Microbiology Research Center, Ilam University of Medical Sciences, Ilam, Iran; Department of Microbiology, Faculty of Medicine, Ilam University of Medical Sciences, Ilam, Iran; Clinical Microbiology Research Center, Ilam University of Medical Sciences, Ilam, Iran; Department of Microbiology, Faculty of Medicine, Ilam University of Medical Sciences, Ilam, Iran; Clinical Microbiology Research Center, Ilam University of Medical Sciences, Ilam, Iran; Department of Microbiology, Faculty of Medicine, Ilam University of Medical Sciences, Ilam, Iran; Clinical Microbiology Research Center, Ilam University of Medical Sciences, Ilam, Iran; Department of Microbiology, Faculty of Medicine, Ilam University of Medical Sciences, Ilam, Iran; Clinical Microbiology Research Center, Ilam University of Medical Sciences, Ilam, Iran; Department of Microbiology, Faculty of Medicine, Ilam University of Medical Sciences, Ilam, Iran; Clinical Microbiology Research Center, Ilam University of Medical Sciences, Ilam, Iran; Department of Microbiology, Faculty of Medicine, Ilam University of Medical Sciences, Ilam, Iran; Clinical Microbiology Research Center, Ilam University of Medical Sciences, Ilam, Iran; Department of Microbiology, Faculty of Medicine, Ilam University of Medical Sciences, Ilam, Iran; Clinical Microbiology Research Center, Ilam University of Medical Sciences, Ilam, Iran; Department of Microbiology, Faculty of Medicine, Ilam University of Medical Sciences, Ilam, Iran; Clinical Microbiology Research Center, Ilam University of Medical Sciences, Ilam, Iran

**Keywords:** P. aeruginosa, antibiotic resistance, mRNA vaccine, epitopes, molecular docking

## Abstract

The rising issue of antibiotic resistance has made treating *Pseudomonas aeruginosa* infections increasingly challenging. Therefore, vaccines have emerged as a viable alternative to antibiotics for preventing *P. aeruginosa* infections in susceptible individuals. With its superior accuracy, high efficiency in stimulating cellular and humoral immune responses, and low cost, mRNA vaccine technology is quickly replacing traditional methods. This study aimed to design a novel mRNA vaccine by using *in silico* approaches against *P. aeruginosa*. The research team identified five surface and antigenic proteins and selected their appropriate epitopes with immunoinformatic tools. These epitopes were then examined for toxicity, allergenicity and homology. The researchers also checked their presentation and identification by major histocompatibility complex cells and other immune cells through valuable tools like molecular docking. They subsequently modeled a multi-epitope protein and optimized it. The mRNA was analyzed in terms of structure and stability, after which the immune system’s response against the new vaccine was simulated. The results indicated that the designed mRNA construct could be an effective and promising vaccine that requires laboratory and clinical trials.

## INTRODUCTION

*Pseudomonas aeruginosa* is a Gram-negative bacillus that causes severe infections in patients with burns, severe wounds and pneumonia, as well as in critically ill patients who require intubation (ventilator-associated pneumonia) or catheterization (urinary tract infections) [1, 2]. The emergence of antibiotic-resistant strains has made treating *P. aeruginosa* infections increasingly difficult [3]. According to the World Health Organization, there is an urgent need for novel therapeutics to combat *P. aeruginosa* infections due to their increasing prevalence and resistance rates. *Pseudomonas aeruginosa* is listed as one of the three bacterial species for which there is the most critical need for developing novel therapeutics [4].

In the USA, multi-drug-resistant (MDR) *P. aeruginosa* caused 32 600 infections among hospitalized patients and an estimated 2700 deaths in 2017 [5]. Resistance rates of *P. aeruginosa* are increasing in many parts of the world, with recent studies reporting the widespread presence of extensively drug-resistant (XDR) high-risk clones in healthcare settings [6, 7]. Therefore, researchers are exploring several evolving strategies for the control and therapy of *P. aeruginosa*, including immunotherapy, phage therapy and vaccination [8]. Vaccines are a promising alternative to antibiotics in preventing *P. aeruginosa* infections in susceptible individuals [9]. They may be the best strategy to overcome treatment-associated complications with MDR *P. aeruginosa*. Several vaccines have entered clinical trials to prevent *P. aeruginosa* infections, but none have been approved for human use [10].

Over the years, vaccine development research has made significant progress. Traditional approaches like living attenuated, inactivated bacteria and subunit vaccines have proven to induce immunogenicity and provide long-term protection [11]. However, novel methods such as peptide-based and DNA vaccines show potential for rapid and scalable vaccine development [12–14]. Unfortunately, peptide-based vaccines have a low immunogenicity index, and DNA vaccines carry the risk of insertional mutagenesis into the host’s DNA. On the other hand, mRNA vaccines are more efficient, addressing the safety and efficacy concerns that arise with DNA and peptide-based vaccines [15, 16]. In addition, cell-free mRNA vaccines can be produced quickly, cost-effectively and at scale. Furthermore, a single mRNA vaccine can encode multiple antigens, enhancing the immune response against resilient pathogens [17].

In designing an effective mRNA vaccine for *P. aeruginosa*, surface proteins critical for binding and entering the bacteria into host cells could serve as suitable targets. The outer membrane of *P. aeruginosa* contains various proteins, including lipoproteins and channels. Porins, which are β-barrel proteins that create water-filled diffusion channels, control nutrient exchange across the outer membrane. Among porin proteins, OprF is a significant target for diagnosing and treating *P. aeruginosa* because it is highly expressed, antigenically conserved and immunogenic [4, 18]. Another essential surface lipoprotein in *P. aeruginosa* pathogenesis is Outer membrane protein I (OprI), which plays a significant role in making the bacteria resistant to antimicrobial peptides. A phase III clinical trial vaccine (NCT01563263) consisting of OprI and OprF proteins shows promise as a vaccine for *P. aeruginosa* [19].

T4 pilli in *P. aeruginosa* are critical in many processes, including attachment to biotic and abiotic surfaces, DNA uptake, biofilm formation, phage transduction, and twitching motility. Therefore, they provide an ideal target antigen for vaccine development [20]. The pilus fiber comprises hundreds of copies of PilA or pilin, which act as both a major structural subunit and an adhesion factor [21]. *Pseudomonas aeruginosa* is equipped with a single polar flagellum, comprising a filament made of helically arranged polymerized flagellin subunits (FliC), a type-specific cap protein (FliD), the hook at the base of the filament (FlgE), two filament-hook junction proteins (FlgKL) and several basal body components across outer and inner membranes [22]. FliC flagellin (paFliC) is crucial for *P. aeruginosa* colonization and acts as an essential virulence factor. It activates innate immune responses by recognizing Toll-like receptor 5 (TLR5) and adaptive immunity in the host. paFliC has been considered a vaccine candidate against *P. aeruginosa* infections [23]. In this study, a novel multi-epitope mRNA vaccine was designed against *P. aeruginosa* using *in silico* approaches.

## MATERIALS AND METHODS

### Retrieval of bacterial protein sequences

The UniProt Knowledgebase (http://www.Uniprot.org) was used to obtain the amino acid sequences of the following proteins: (1) fliC (accession number P21184), (2) fliD (accession number O33421), (3) oprI (accession number P11221), (4) oprF (accession number P13794) and (5) pilA (accession number P04739).

### Prediction of immune cell epitopes

To predict B-cell epitopes, the ABCpred webserver available at https://webs.iiitd.edu.in/raghava/abcpred/ABC was used. An artificial machine-learning approach was employed for epitope prediction, with each protein sequence submitted using a 0.5 threshold. The selected epitope length was 16 amino acids, and the overlap filter remained active. The top epitope results were further investigated [24].

In addition, cytotoxic T-cell lymphocyte (CTL) epitopes were predicted using the ANN 4.0 method through the Immune Epitope Database MHC, which can be accessed at http://tools.iedb.org/main/tcell/. Predicted epitopes were sorted based on their IC_50_ value. Helper T-cell lymphocytes were predicted using the NN-align method through the Immune Epitope Database MHC-II, which can be accessed at http://tools.iedb.org/main/tcell/ [25].

### Human homology

To check for homology between the predicted peptides and human peptides, the NCBI BLASTp tool available at https://blast.ncbi.nlm.nih.gov/Blast.cgiPAGE=Proteins was used to compare all peptides against the *Homo sapiens* (TaxID: 9606) protein database. Peptides with an E-value greater than 0.05 were considered possibly non-homologous to human peptides.

### Prediction of epitope’s antigenicity, allergenicity and toxicity

To evaluate the antigenicity, allergenicity and toxicity of selected epitopes, various web servers were used. The VaxiJen web server available at http://www.ddg-pharmfac.net/Vaxijen/VaxiJen/VaxiJen.html predicted antigenicity based on the physicochemical properties of the epitopes in an alignment-independent manner, with a focus on bacteria and a threshold of 0.4. The AllerTop V.2.0 webserver at http://www.ddg-pharmfac.net/AllerTOP was used to predict allergenicity of epitopes using default settings. Lastly, the ToxinPred server available at https://webs.iiitd.edu.in/raghava/toxinpred/multi_submit.php predicted and measured toxicity of epitopes by generating all potential mutants using default settings. Only epitopes that were antigenic, non-toxic and non-allergenic were retained for further research.

### Molecular docking between T-lymphocyte epitopes and MHC alleles

Molecular docking simulations were used to evaluate the binding affinity of selected T-lymphocyte epitopes to their corresponding major histocompatibility complex (MHC) alleles. The 3D structures of MHC alleles were obtained from the RCSB PDB database and processed using PyMOL software to remove unnecessary ligands. Energy minimization of the structures was performed using Swiss-PDB Viewer. The selected epitopes were folded into their respective three-dimensional structures using the PEP-FOLD 3.5 server and then energy minimized using Swiss-PdBViewer before docking. Docking was performed using the ClusPro 2.0 server available at https://cluspro.bu.edu/login.php.

### Design of the vaccine construct

A proposed mRNA vaccine construct has been designed using a specific sequence order from the N to C terminus. The sequence includes a modified cap structure (m7GCap), followed by a 5′ untranslated region (5′UTR) and a Kozak sequence to enhance translation. The coding region begins with a signal peptide (tPA) connected through an EAAAK linker to an adjuvant component (RpfE), separated by a GPGPG linker. The vaccine’s epitopes have been grouped into three sets and linked together via AAY, KK and GPGPG linkers, which provide cleavability, flexibility, rigidity, and separate domains for proper folding and functioning of the components. The HTL epitopes are connected to the LBL epitopes using the KK linker, while the LBL epitopes are connected to the CTL epitopes via an AAY linker. The mRNA construct terminates with a MITD sequence, followed by a stop codon, a 3′UTR and a poly(A) tail to ensure proper termination and stability of the mRNA molecule. This sequence offers a promising approach to developing vaccines against certain diseases.

### Prediction of antigenicity, allergenicity, toxicity and physicochemical properties of the vaccine construct

To determine the antigenicity of the mRNA vaccine construct, the VaxiJen 2.0 and ANTIGENpro servers were utilized. VaxiJen 2.0 predicts based on the physicochemical properties of the vaccine, while ANTIGENpro uses machine-learning algorithms and microarray analysis data. The constructed mRNA vaccine’s amino acid sequence were used as the input, excluding tPA and MITD sequences. To assess allergenicity, the AllerTOP 2.0 server was used, and the ToxinPred server predicted toxicity. Finally, the ProtParam online web server (https://web.expasy.org/protparam/) was employed to predict various physicochemical properties of the vaccine, including amino acid composition, molecular weight, theoretical isoelectric point (p*I*), instability index (II), aliphatic index (A.I.) and grand average of hydropathicity (GRAVY).

### *In silico* immune simulation

The C-ImmSim online simulation server (http://150.146.2.1/CIMMSIM/index.php) was utilized to simulate the immune response for the mRNA vaccine construct. An immune response is stimulated by this server using epitopes in conjunction with lymphocyte receptors. To simulate the recommended dosage schedule of current vaccines, three doses of 1000 vaccine units were administered over 4 weeks. For the purposes of this study, all parameters were set to default values, and the injections were administered at time-steps 1, 84 and 168. By conducting a dynamic simulation of the immune response, the performance of the mRNA vaccine construct and its potential efficacy in eliciting an immune response can be assessed by researchers.

### Codon optimization of the vaccine construct

The codon sequences in the designed mRNA vaccine construct were optimized to ensure efficient expression within human cells. For this purpose, the GenSmart Codon Optimization Tool (http://www.genscript.com/) provided by GenScript (G.S.) was used. After optimization, a quality assessment of the optimized sequence was performed using the Rare Codon Analysis tools (http://www.genscript.com/) also provided by GenScript. The efficiency of mRNA translation was determined using Codon Adaptation Index (CAI). In addition, any unusual tandem codons present in the optimized sequence were identified through codon frequency distribution analysis. By optimizing the codon sequences, the expression and efficacy of the mRNA vaccine construct can potentially be improved by researchers.

### Secondary structure prediction of the designed mRNA vaccine

The RNAfold tool (http://rna.tbi.univie.ac.at/cgi-bin/RNAWebSuite/RNAfold.cgi), which is part of the Vienna RNA Package 2.0, was used to determine the predicted secondary structure of the mRNA vaccine construct. McCaskill’s algorithm was utilized by the RNAfold tool to calculate the minimum free energy (MFE) of the predicted secondary structure. Through this tool, researchers obtained information on both the minimal free energy structure and the centroid secondary structure, as well as their respective minimum free energies. By predicting the secondary structure of the mRNA vaccine construct, researchers can gain a better understanding of its potential stability and functionality within human cells.

### Prediction and validation of the secondary and tertiary structures of the designed vaccine

The PSIPRED server (http://bioinf.cs.ucl.ac.uk/psipred/) was used by researchers to predict the secondary structure of the peptide sequence in the mRNA vaccine construct, excluding the tPA signal and MITD sequences. This tool employs position-specific scoring matrices and has an accuracy rate of 84.2%. To predict the three-dimensional structure of the peptide sequence, the Robetta server (https://robetta.bakerlab.org/) was utilized, which generated five possible structures. The ProSA-web (https://prosa.services.came.sbg.ac.at/prosa.php), PROCHECK and ERRAT (https://saves.mbi.ucla.edu/) servers were then used to verify the best structure. By predicting both the secondary and tertiary structures of the peptide sequence, researchers can gain a better understanding of its potential function within the mRNA vaccine construct.

### Prediction of the conformational B-cell epitopes

ElliPro, an online server (http://tools.iedb.org/ellipro/), was employed to identify discontinuous B-cell epitopes within the studied protein structure. ElliPro uses the geometrical characteristics of the 3D model to predict these epitopes. Compared to other available prediction tools for discontinuous B-cell epitopes, ElliPro provides the highest AUC value of 0.732 for any protein model. By identifying these epitopes, researchers can gain a better understanding of the potential immunogenicity of the protein structure and its role in the overall efficacy of the mRNA vaccine construct. The identification of these epitopes can also aid in the development of future vaccines and immunotherapies.

### Molecular docking of the designed vaccine

The ClusPro server was utilized to determine the potential interaction between the desired mRNA vaccine and toll-like receptor 4 (TLR-4) or toll-like receptor 3 (TLR-3). The 3D structures of both the vaccine and TLR-4 (PDB ID: 3FXI) or TLR-3 (PDB ID: 1ZIW) were docked using the PIPER docking algorithm. By predicting how the vaccine structure interacts with TLR-4 or TLR-3, researchers can gain a better understanding of the potential immunogenicity of the vaccine construct and its overall efficacy in eliciting an immune response.

### Molecular dynamics simulation

To confirm the physical motions of atoms and molecules within the TLR4-vaccine and TLR3-vaccine complex structures, dynamics simulation analysis was conducted using the iMODS server (http://imods.chaconlab.org/). The complex structures with the lowest binding energy were utilized for this analysis to ensure accuracy. The movement of atoms and molecules within the complex structures over time was simulated and their stability was assessed through the iMODS server. By understanding the dynamic behavior of the complex structures, insight into their potential efficacy in eliciting an immune response can be gained by the researchers.

## RESULTS

### Prediction and estimation of B-cell epitopes

In this study, the selection of epitopes was limited to the top five predicted for each included protein by the ABCpred webserver. The selected epitopes were subjected to further filtering to ensure their antigenicity, non-allergenicity and non-toxicity, using the VaxiJen, AllerTop and ToxinPred web servers, respectively. To avoid the induction of autoimmunity by the vaccine construct, all predicted epitopes were screened for homologs among *Homo sapiens* (Taxid:9606) with an E-value ˂0.05 and any homologs found were excluded from the vaccine construct. The selected protein variants were downloaded from the NCBI database and aligned using the Bioedit 7.2 program. In total, five B-cell epitopes extracted from the five studied proteins were chosen for inclusion in the vaccine construct, as listed in Table 1.

**Table 1:** Cell type and sequence of epitope in this study

Cell type	Sequence of epitope
CD8^+^ cytotoxic T lymphocytes	KMDGAIPNL
	VQLNSPTAY
	NIASLNTQR
	DAAGLQISNR
	LSDGAAAGY
	AALGITTTK
	SADFEGVASY
	GTYETGNKK
	RGTYETGNKK
	TTVEESLSR
	LAAIAIPQY
CD4^+^ helper T lymphocytes	QSANGSNSDADRAAL
	ANGSNSDADRAALQK
	NGSNSDADRAALQKE
	QSANGSNSDADRAAL
	SANGSNSDADRAALQ
	SDDGSGGNTSLSQLA
	EVSDDGSGGNTSLSQ
	KVEVSDDGSGGNTSL
	LSSTKTGDGKDIKVE
	AAPAPEPVADVCSDS
	GGSKAAPAPEPVADV
	GSKAAPAPEPVADVC
	KAAPAPEPVADVCSD
	GAAQKAQQTADEANE
	SKETEARLTATEDAA
	ASTATETYVGVEPDA
	EDSGAGDITFTFQTG
	TTASTATETYVGVEP
B Lymphocyte	ASAIGSYQVGSNGAGT
	TVSRDDAGVKDNVKKF
	RGTYETGNKKVHGNLT
	AGLGVGFNFGGSKAAP
	TGCSSHSKETEARLTA

### Prediction and estimation of the CTL epitopes

In this study, the researchers used the IEDB database to identify potential cytotoxic T lymphocyte (CTL) epitopes from the nine proteins studied. Epitopes with an IC_50_ over 500 were selected for further analysis. Only epitopes that were antigenic, non-allergenic, non-toxic and non-homologs were included in the subsequent analysis. From these selection criteria, 11 epitopes located in the conserved regions of the proteins were chosen for inclusion in the vaccine construct. By selecting conserved epitopes, the researchers aimed to create a vaccine with broad efficacy against multiple strains of the target virus or pathogen. The selected epitopes are listed in Table 1.

### Prediction and estimation of the HTL epitopes

Several potential HTL epitopes were identified from studying five *P. aeruginosa* proteins mentioned previously. Epitopes that were antigenic, non-allergenic, non-toxic, and non-homologs were investigated for their ability to induce cytokines, specifically IL-4, IL-10 and IFN-γ. From these analyses, 18 epitopes located in the conserved regions of the proteins were chosen for inclusion in the vaccine construct. These epitopes induced the cytokines mentioned earlier and are listed in Table 1. By selecting epitopes that induce cytokine responses, the researchers aimed to enhance the immune response elicited by the vaccine construct.

### Molecular docking between MHC alleles and the selected T-lymphocyte epitopes

**Figure 1:**
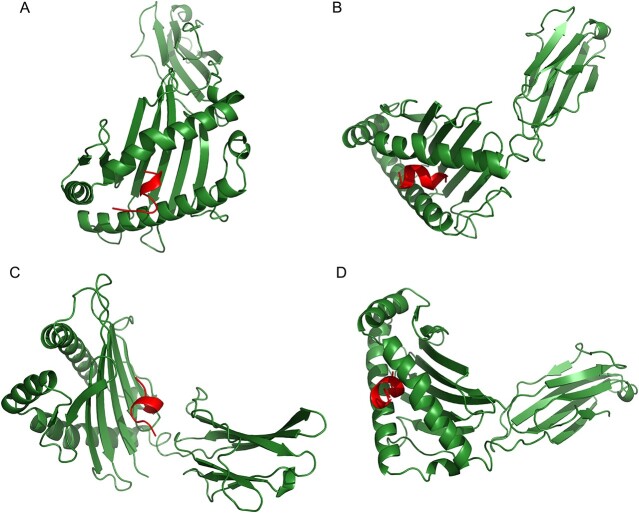
Docking between the epitopes and their corresponding MHC allele.

**Figure 2:**
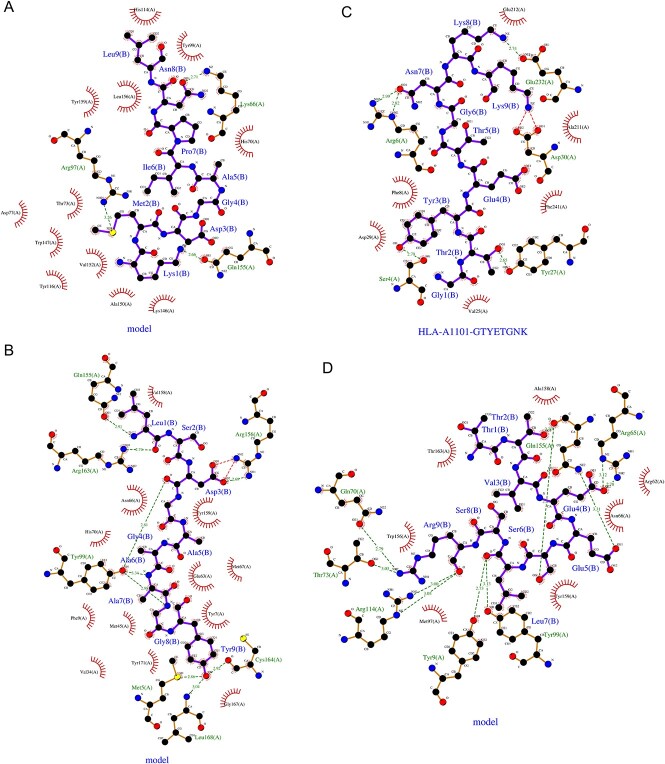
Epitopes and their corresponding MHC allele interaction using the LIGPLOT webserver.

In this study, the researchers identified 29 lymphocyte epitopes that recognized a total of 38 MHC alleles, with some epitopes binding to one allele and others binding to multiple alleles (Table 2). Four epitopes and their corresponding MHC alleles were selected for further molecular docking analysis, with the crystallographic structures of all MHC alleles selected from the RCSB PDB server (Table 2). Using ClusPro 2.0, molecular docking was performed on these four epitopes and their parallel MHC alleles, and the results are presented in Table 3. The epitope LSDGAAAGY displayed the strongest binding affinity with its corresponding MHC allele (HLA-A*01:01) with a −643.6 kcal/mol value. Furthermore, it was found that this epitope fits perfectly inside the binding cleft of its corresponding allele (Figure 1B). Finally, the interactions between the epitope and various residues of the MHC allele were evaluated using the LIGPLOT website (Figure 2).

**Table 2:** Selected T-lymphocyte epitopes and their corresponding MHC alleles’ protein name

Protein name	Function	CTL epitopes	MHC I binding alleles	HTL epitopes	MHC II binding alleles
FliC	A-type flagellin	KMDGAIPNL	HLA-A*02:01	QSANGSNSDADRAAL	HLA-DRB1*15:01
		VQLNSPTAY	HLA-B*15:01	ANGSNSDADRAALQK	HLA-DRB1*15:01
		NIASLNTQR	HLA-A*68:01	NGSNSDADRAALQKE	HLA-DRB1*15:01
		DAAGLQISNR	HLA-A*02:06	QSANGSNSDADRAAL	HLA-DRB1*07:01 HLA-DRB5*01:01
				SANGSNSDADRAALQ	HLA-DRB1*15:01
FliD	Full = flagellar cap protein	LSDGAAAGY	HLA-A*01:01	SDDGSGGNTSLSQLA	HLA-DRB1*03:01 HLA-DRB5*01:01
		AALGITTTK	HLA-A*11:01	EVSDDGSGGNTSLSQ	HLA-DRB1*15:01 HLA-DRB4*01:01 HLA-DRB5*01:01
		SADFEGVASY	HLA-A*01:01		
				KVEVSDDGSGGNTSL	HLA-DRB5*01:01
				LSSTKTGDGKDIKVE	HLA-DRB1*15:01
OprF	Major outer membrane protein	GTYETGNKK	HLA-A*11:01	AAPAPEPVADVCSDS	HLA-DRB5*01:01
		RGTYETGNKK	HLA-A*03:01	GGSKAAPAPEPVADV	HLA-DRB1*03:01
				GSKAAPAPEPVADVC	HLA-DRB1*03:01
				KAAPAPEPVADVCSD	HLA-DRB5*01:01
OprI	Major outer membrane lipoprotein I			GAAQKAQQTADEANE	HLA-DRB1*03:01 HLA-DRB1*15:01 HLA-DRB3*02:02
				SKETEARLTATEDAA	HLA-DRB5*01:01 HLA-DRB1*15:01
PilA	Fimbrial protein	TTVEESLSR	HLA-A*68:01	ASTATETYVGVEPDA	HLA-DRB5*01:01 HLA-DRB1*03:01 HLA-DRB1*15:01
		LAAIAIPQY	HLA-B*35:01	EDSGAGDITFTFQTG	HLA-DRB5*01:01
				TTASTATETYVGVEP	HLA-DRB5*01:01

**Table 3:** Docking analysis of some CTL epitopes with their corresponding MHC alleles

Type of T lymphocyte	Epitope	MHC alleles	PDB ID of MHC allele	Binding affinity (kcal/mol)
CTL	KMDGAIPNL	HLA-A*02:01	4U6X	−550.2
	LSDGAAAGY	HLA-A*01:01	6MPP	−643.6
	GTYETGNKK	HLA-A*11:01	7S8Q	−447.8
	TTVEESLSR	HLA-A*68:01	6PBH	−569.0

### Vaccine construct design

The mRNA vaccine construct was proposed to be arranged from the N to C terminus in the following order: 5′ m7GCap, 5′ UTR, Kozak sequence, Signal peptide (tPA), EAAAK Linker, Adjuvant (RpfE)–GPGPG Linker–QSANGSNSDADRAAL–GPGPG Linker–ANGSNSDADRAALQK–GPGPG Linker–NGSNSDADRAALQKE–GPGPG Linker–QSANGSNSDADRAAL–GPGPG Linker–SANGSNSDADRAALQ–GPGPG Linker–SDDGSGGNTSLSQLA–GPGPG Linker–EVSDDGSGGNTSLSQ–GPGPG Linker–KVEVSDDGSGGNTSL–GPGPG Linker–LSSTKTGDGKDIKVE–GPGPG Linker–AAPAPEPVADVCSDS–GPGPG Linker–GGSKAAPAPEPVADV–GPGPG Linker–GSKAAPAPEPVADVC–GPGPG Linker–KAAPAPEPVADVCSD–GPGPG Linker–GAAQKAQQTADEANE–GPGPG Linker–SKETEARLTATEDAA–GPGPG Linker–ASTATETYVGVEPDA–GPGPG Linker–EDSGAGDITFTFQTG–GPGPG Linker–TTASTATETYVGVEP–GPGPG Linker–ASTATETYVGVEPDA–KK Linker–ASAIGSYQVGSNGAGT–KK Linker–TVSRDDAGVKDNVKKF–KK Linker–RGTYETGNKKVHGNLT–KK Linker–AGLGVGFNFGGSKAAP–KK Linker–TGCSSHSKETEARLTA–AAY Linker–KMDGAIPNL–AAY Linker–VQLNSPTAY–AAY Linker–NIASLNTQR–AAY Linker–DAAGLQISNR–AAY Linker–LSDGAAAGY–AAY Linker–AALGITTTK–AAY Linker–SADFEGVASY–AAY Linker–GTYETGNKK–AAY Linker–RGTYETGNKK–AAY Linker–TTVEESLSR–AAY Linker–LAAIAIPQY–AAY Linker–MITD sequence–Stop codon–3′ UTR–Poly (A) tail.

### Evaluation of antigenicity, allergenicity, toxicity and physicochemical properties of the vaccine construct

To assess the antigenicity, allergenicity and toxicity of the vaccine construct, the VaxiJen, ANTIGENpro, AllerTop and ToxinPred servers were utilized by the researchers. In addition, the physicochemical properties of the vaccine were evaluated using the ProtParam server (Table 4). The results indicated that the vaccine construct was found to be antigenic, non-allergenic and non-toxic. Furthermore, the physicochemical properties of the vaccine suggested that it was thermally stable, with a GRAVY score of −0.592, indicating its hydrophilic nature. Based on these findings, the multi-epitope mRNA vaccine construct developed in this study has the potential to be an effective candidate against *P. aeruginosa*.

**Table 4:** The physicochemical properties of the translated form of the proposed mRNA vaccine

Property	Measurement	Indication
Total number of amino acids	600	Appropriate
Molecular weight	57 929/33	Appropriate
Formula	C2463H3861N719O889S5	–
Theoretical p*I*	4.73	Acidic
Total number of negatively charged residues (Asp + Glu)	2	–
Total number of positively charged residues (Arg + Lys)	47	–
Total number of atoms	7937	–
Instability Index (II)	23.52	Stable
Aliphatic Index (A.I.)	49.87	Thermostable
Grand average of hydropathicity (GRAVY)	−0.592	Hydrophilic
Antigenicity (using VaxiJen)	1.3278	Antigenic
Antigenicity (using ANTIGENpro)	0.909268	Antigenic
Allergenicity (using AllerTop 2.0)	Non-allergenic	Non-allergenic
Toxicity (ToxinPred)	Non-toxic	Non-toxic

### Population coverage prediction

The IEDB Population Coverage tool was used to measure the global population coverage of the 29 epitopes for their corresponding 38 alleles. The results indicated that the vaccine construct has the potential to cover ~90.13% of the world’s population, suggesting that it could provide broad protection against *P. aeruginosa* infections.

### *In silico* immune response simulation against the vaccine

**Figure 3:**
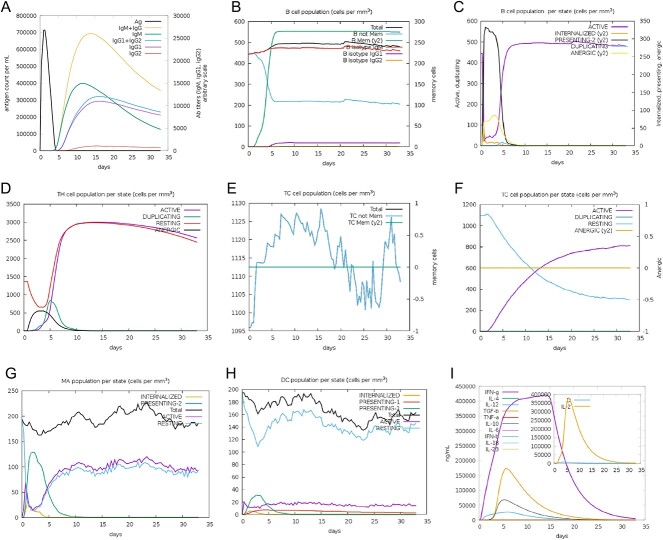
*In silico* immune simulation against the mRNA vaccine retrieved from the C-ImmSim server (https://kraken.iac.rm.cnr.it/C-IMMSIM/). (**A**) The immunoglobulin production after antigen injection. (**B**) The B-cell population after three injections. (**C**) The B-cell population per state. (**D**) The helper T-cell population. (**E**) The helper T-cell population per state. (**F**) The cytotoxic T-cell population per state. (**G**) Macrophage population per state. (**H**) Dendritic cell population per state. (**I**) Cytokine and interleukin production with Simpson Index of the immune response.

In the study, three vaccine injections were administered to simulate the immune response (Figure 3). The second and third injections elicited higher immune responses compared to the primary injection. Immunoglobulin M (IgM) levels were higher than IgG levels, and the levels of immunoglobulins remained elevated following antigen reduction, suggesting the potential emergence of immune memory and practiced immunity. Isotype switching and memory formation of the B-cell population were also observed, with the presence of B-cell isotypes persisting for a prolonged duration. In addition, an increase in CTL and HTL cells with memory generation was observed. Furthermore, macrophage activity was enhanced, while dendritic cell activity remained stable. Levels of IFN-γ and IL-2 cytokines were also increased. Epithelial cells, which are components of innate immunity, were augmented as well. Lastly, the Simpson index (D) was low, indicating a diverse immune response. Overall, these results suggest that the multi-epitope mRNA vaccine construct elicits a robust and diverse immune response against *P. aeruginosa*.

### Codon optimization of the mRNA construct

**Figure 4:**
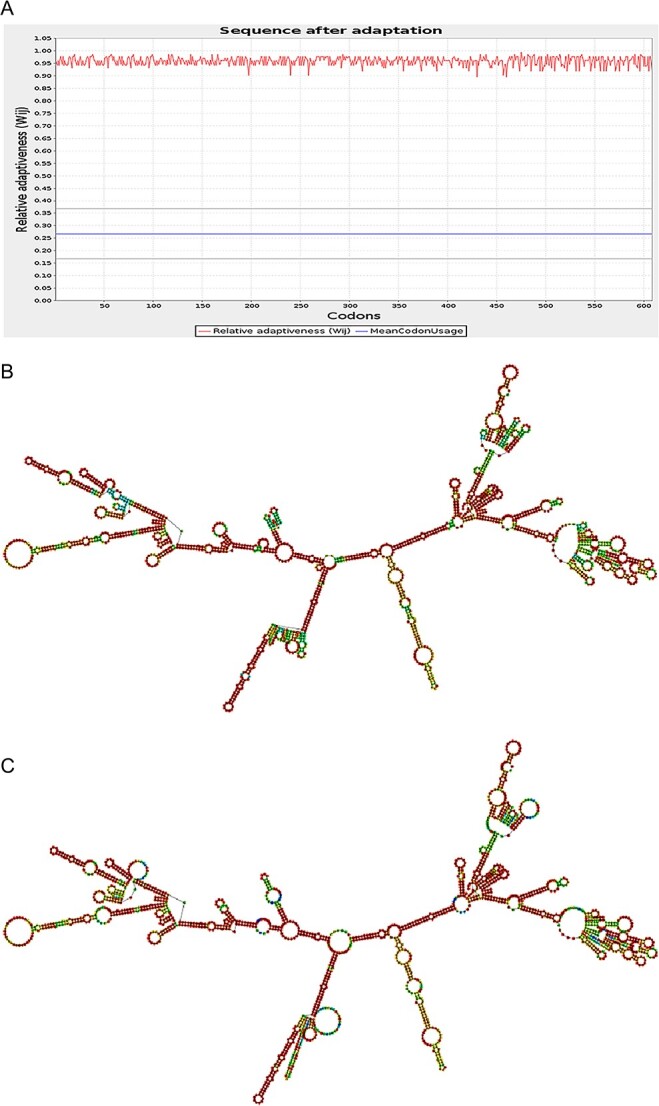
Codon optimization and mRNA vaccine structure prediction: (**A**) CAI value; (**B**) optimal secondary structure; (**C**) centroid secondary structure of the vaccine mRNA retrieved using RNAfold webserver.

To enhance the translation of the mRNA vaccine construct within host cells, codon optimization tools were utilized. The GenSmart Codon Optimization tool (G.S.) was used to optimize the vaccine sequence for efficient expression in human cells. The length of the CDS was 1824 nucleotides, and the rare codon analysis tool from G.S. was employed to evaluate the quality of the optimized construct. The CAI value was estimated to be 0.96 (Figure 4A), which is acceptable since it exceeds the cut-off value of 0.8. In addition, the optimized construct’s G.C. content was evaluated, and it was found that the optimal percentage of G.C. content should be ~30–70% to ensure efficient expression in the human host. The average G.C. percentage of the optimized construct was 76.53%. This indicates that no codons could impede translation efficiency or function, as any codons with a value lower than 30 could reduce or stop translational machinery. Overall, these analyses suggest that the codon-optimized mRNA vaccine construct can be efficiently expressed in human cells.

### Prediction of the secondary structure of the mRNA vaccine

The RNAfold server was used to predict the structure of the mRNA vaccine construct. The optimized codons of the construct were used as input, and the free energies of the structure were assessed using the server. The results indicated that the mRNA vaccine construct would be stable when manufactured with the MFE of the structure, which was calculated to be −739.10 kcal/mol (Figure 4B). In addition, the secondary centroid structure had a free energy of −680.30 kcal/mol (Figure 4C). These findings suggest that the mRNA vaccine construct can be efficiently manufactured and is structurally stable, potentially enhancing its efficacy as a vaccine.

### Prediction and validation of the secondary and tertiary structures of the translated construct

**Figure 5:**
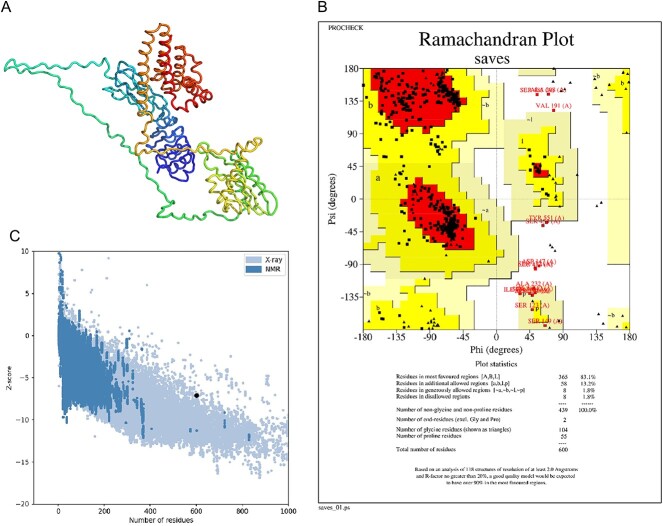
Structure prediction and validation of the peptide vaccine construct: (**A**) tertiary structure of the peptide using the Robetta server; (**B**) Ramachandran plot analysis using the PROCHECK server; (**C**) *Z*-score analysis using ProSA webserver.

The PSIPRED web service was used by researchers to further analyze the structure of the mRNA vaccine construct and predict its secondary structure. The alpha helices that predominated in the structure are shown in Figure 5A (Supplementary file 1). In addition, the Robetta server was used to predict the tertiary structure of the peptide (Figure 5B), and the PROCHECK server was employed to verify the stereochemical accuracy of the structure. The Ramachandran plot presented in Figure 5C indicated that ~83.1% of the residues were in the most favored regions, 13.2% in the additionally allowed zone and 1.8% in the generously allowed regions. The overall quality factor was 93.5275. Moreover, a negative Z-score (−7.09) was predicted by the ProSA-web, indicating that the 3D protein model is highly consistent (Figure 5D). Overall, these analyses suggest that the structure of the mRNA vaccine construct is stable, accurate and consistent, potentially enhancing its efficacy as a vaccine.

### Conformational B-cell epitope prediction

**Figure 6:**
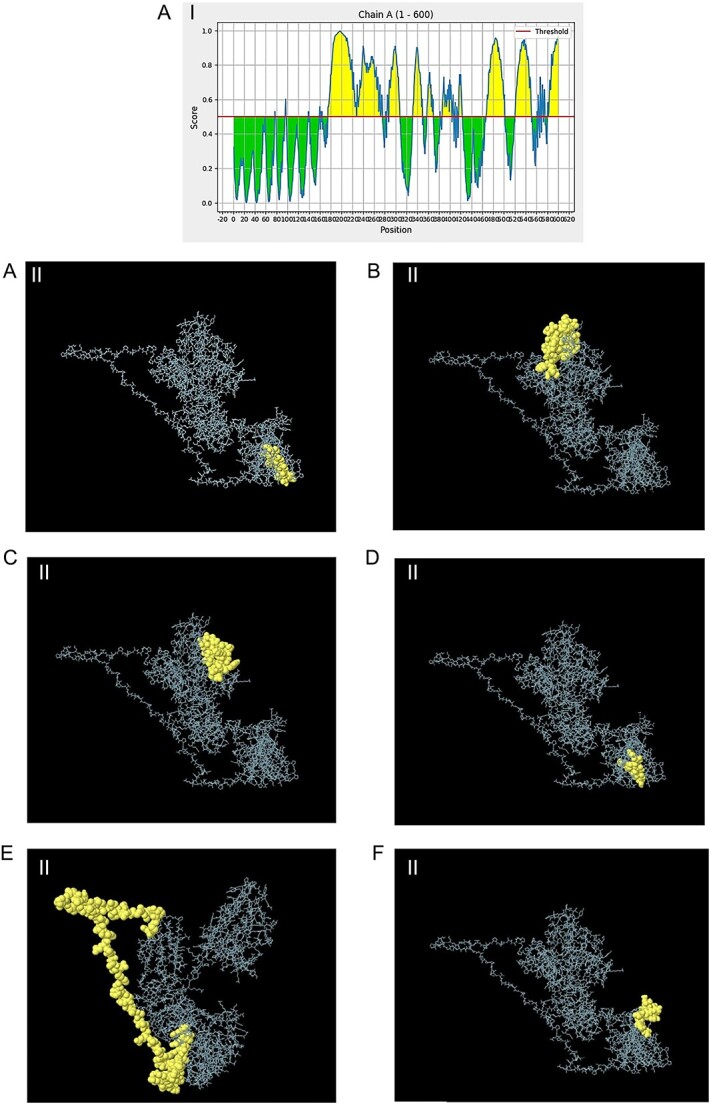
The six predicted conformational B-cell epitopes using the ElliPro tool of the IEDB database: (I) 2D diagra m of the positions of conformational B-cell epitopes. (II) The 3D models of B-cell epitopes. The spheres represent the conformational B-cell epitopes. (**A**) 14 residues with a score of 0.613. (**B**) 37 residues with a score of 0.779. (**C**) 32 residues with a score of 0.791. (**D**) 120 residues with a score of 0.681. (**E**) 7 residues with a score of 0.582. (**F**) 5 residues with a score of 0.515.

The ElliPro server was used by researchers to predict the conformational B-cell epitopes generated from the folding of the model protein. The results revealed the prediction of six discontinuous B-cell epitopes, with a prediction score ranging from 0.515 to 0.791 for 215 residues. The 2D and 3D models of these conformational B-cell epitopes are shown in Figure 6I and II, respectively. These findings suggest that strong B-cell immune responses against *P. aeruginosa* infections can potentially be elicited by the mRNA vaccine construct.

### Molecular dynamics simulation

**Figure 7:**
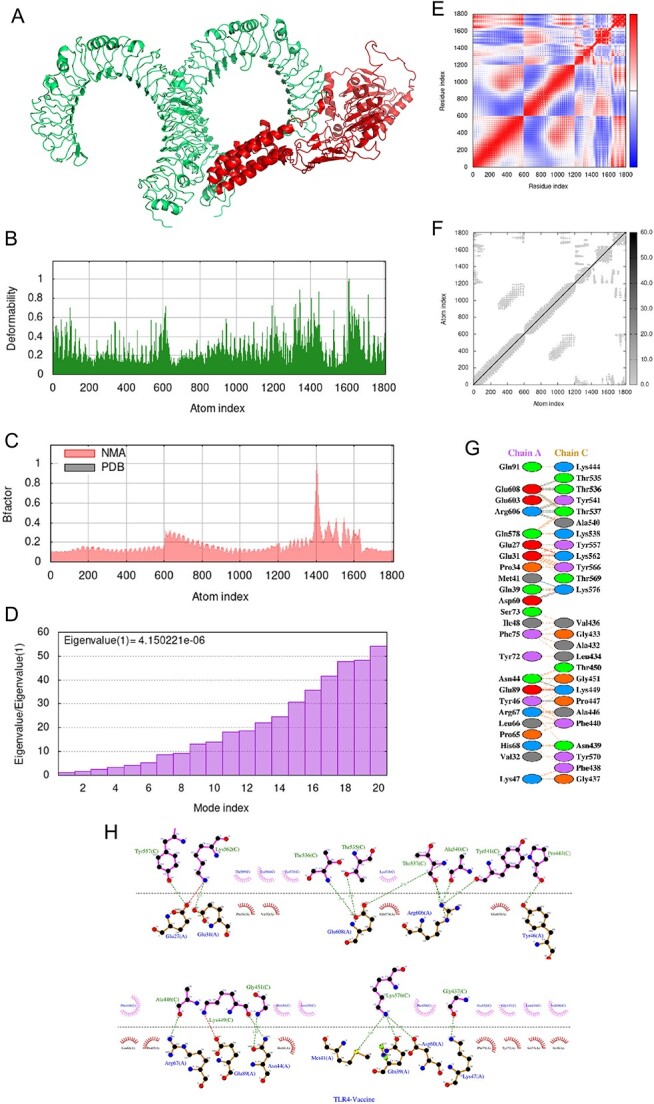
Molecular dynamics simulation, normal mode analysis and receptor–ligand interactions: (**A**) vaccine-TLR4 docked complex using the Cluspro server; (**B**) deformability graph; (**C**) B-factor graph; (**D**) eigenvalue of vaccine-TLR4 complex; (**E**) covariance matrix; (**F**) elastic network model using the iMODS server; (**G**) receptor–ligand interaction using the PDBsum webserver; (**H**) receptor–ligand interaction using the LIGPLOT webserver.

**Figure 8:**
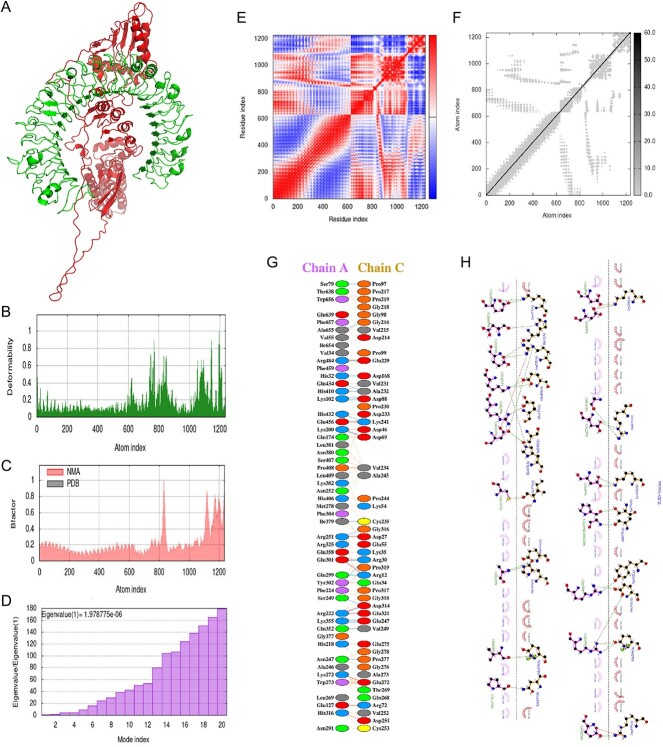
Molecular dynamics simulation, normal mode analysis and receptor–ligand interactions: (**A**) vaccine-TLR3 docked complex using the Cluspro server; (**B**) deformability graph; (**C**) B-factor graph; (**D**) eigenvalue of vaccine-TLR3 complex; (**E**) covariance matrix; (**F**) elastic network model using the iMODS server; (**G**) receptor–ligand interaction using the PDBsum webserver; (**H**) receptor–ligand interaction using the LIGPLOT webserver.

To further analyze the vaccine-TLR3 and vaccine-TLR4 complexes, molecular dynamics simulation was performed using the iMODS server, while receptor–ligand interactions were assessed. The deformable loci of the construct were represented by peaks in the deformability graph (Figures 7B and 8B), which showed amino acids with coiled shapes. Normal mode analysis (NMA) was also conducted to study and characterize protein flexibility, with the B-factor graph (Figures 7C and 8C) depicting the relationship between NMA and PDB areas in the uploaded complex. Eigenvalues of the docked complexes are displayed in Figures 7D and 8D. Overall, these analyses indicated that the vaccine-receptor complex had a low deformation index, muscular stiffness and high stability.

In Figures 7E and 8E, the covariance matrix showed the connection between amino acid duplets scattered in dynamical regions, with red indicating correlated residues, white representing anti-correlated amino acid duplets and blue representing non-correlated residues. In addition, a connecting matrix representing the elastic network model was employed to classify which atom pairs were connected by springs (Figures 7F and 8F). Each chain of the complex was found to have high stiffness, with darker gray colors indicating stiffer regions. These findings suggest that the vaccine-receptor complex is stable, rigid and has strong intermolecular interactions, potentially enhancing its efficacy as a vaccine against *P. aeruginosa*.

## DISCUSSION

*Pseudomonas aeruginosa* is a challenging pathogen due to its antibiotic resistance, and developing effective vaccines against it has become crucial [26]. Research on *P. aeruginosa* vaccines has been ongoing for over half a century, but despite extensive efforts, there are still no approved vaccines to date [27]. The complexity of *P. aeruginosa*’s pathogenesis, diverse virulence factors, high plasticity within the lung and high diversity of serotypes are significant obstacles in developing an effective vaccine [28]. Both innate and adaptive immune responses play critical roles in combating *P. aeruginosa* infection. As *P. aeruginosa* is an extracellular pathogen, humoral, mucosal or systemic opsonizing immunity is most effective in preventing bacterial colonization and infection. However, T-cell responses can also mediate protective immunity in individuals with *P. aeruginosa* infections [29]. Despite the challenges posed by the emergence of MDR and XDR strains, complex pathogenesis, high serotype diversity, and more, continued research and collaboration among scientists may lead to the development of an effective vaccine to combat this critical health challenge [30].

Immunoinformatic approaches were used in a study to develop a novel mRNA vaccine that is safe, engineered and efficient. This mRNA-based vaccine has been shown to be effective against various viral infections such as Zika, influenza, rabies, coronavirus and many others [31]. Recently, multiple human clinical trials have begun, indicating that mRNA vaccines are now considered to be a safe and effective alternative to subunit protein, chimeric virus and even DNA-based therapies in the form of vaccination [32]. One of the benefits of mRNA-based vaccines is their ability to induce the transient expression and accumulation of selected antigens in the cytoplasm, which then triggers an immune response against the target pathogen [33]. This approach may offer several advantages over traditional vaccine strategies, including ease of production, rapid development and improved efficacy.

The development of a safe and efficient mRNA vaccine using immunoinformatic approaches represents a promising advancement in the field of vaccination. With increasing preclinical evidence and ongoing clinical trials, mRNA vaccines may become a preferred option for preventing viral infections and related diseases. In this study, a novel *in silico* multi-epitope mRNA vaccine has been proposed to combat the infection crises caused by *P. aeruginosa*. The vaccine is based on the major surface proteins of *P. aeruginosa* that contribute to cell binding and attachment of the bacterium. This approach may offer a potential solution to the challenges posed by *P. aeruginosa* infections, and further research is needed to evaluate the safety and efficacy of this vaccine. To identify potential epitopes that could induce humoral or cellular responses, the target proteins were examined using web-based tools such as the IEDB database, which predicts epitopes of HTL and CTL based on immune epitope determination, and ABC pred, an online server that anticipates B-cell epitopes using an artificial machine-learning method. The evaluation of epitopes was performed using web servers to determine antigenicity, allergenicity and toxicity. Specific linkers were used to combine the epitopes.

To further refine the vaccine design, an immune simulation was conducted to validate the humoral and cellular responses of the vaccine. The vaccine construct’s targeted epitopes had 38 corresponding MHC alleles. The four chosen epitopes were subjected to molecular docking analysis, as ligand–epitope interaction is essential in vaccine design. Docking analysis was performed among the chosen epitopes and their corresponding MHC alleles using ClusPro, which predicts binding affinity and bond formation between the receptor and ligands. The interactions among the epitopes and MHC pockets were also analyzed. These steps were taken to ensure that the vaccine design was optimized for maximum efficacy.

This study proposes a novel *in silico* multi-epitope mRNA vaccine against *P. aeruginosa* based on major surface proteins of the bacterium. Various bioinformatics tools were utilized to predict and evaluate epitopes, and immune simulations were conducted to validate the vaccine’s effectiveness. The results of molecular docking analysis suggest strong binding affinity between the chosen epitopes and their corresponding MHC alleles, indicating the potential efficacy of the proposed vaccine. It is important to note that vaccination is only effective in individuals with a particular MHC allele that can bind the epitope. Therefore, the IEDB population coverage tool was used to predict that the proposed vaccine would cover 90.13% of the world’s population. This indicates that the vaccine has the potential to be widely effective. Moreover, to evaluate the vaccine’s capacity to interact with immune receptors, the TLR-3 and TLR-4 immune receptors were docked with the vaccine construct. The findings showed that the vaccine has a strong affinity for binding to TLR-4 and TLR-3, indicating the potential for triggering both innate and adaptive immunity. This is a promising result as it suggests that the vaccine has the potential to stimulate a robust immune response. Overall, the proposed vaccine shows great potential for providing protection against *P. aeruginosa* infection.

The stability of the vaccine complex was investigated using molecular dynamics simulation, which showed that the proposed mRNA vaccine’s peptide sequence is stable and thermostable. Immunoinformatic approaches were also used to evaluate the vaccine’s antigenicity, allergenicity and hydrophilicity, and the results indicate that the vaccine is antigenic, non-allergenic and hydrophilic. In this study, the proposed *in silico* multi-epitope mRNA vaccine against *P. aeruginosa* has been evaluated for its potential effectiveness in triggering an immune response. The predicted population coverage is high, and the vaccine construct has a strong affinity for binding to immune receptors TLR-4 and TLR-3. In addition, molecular dynamics simulations indicate that the vaccine complex is stable, antigenic and non-allergenic. These findings suggest that the proposed vaccine may be a promising approach to combat *P. aeruginosa* infections. Overall, the study provides valuable insights into the development of effective vaccines against *P. aeruginosa* and highlights the potential of *in silico* approaches for vaccine design.

## CONCLUSION

In conclusion, the proposed design of a novel multi-epitope mRNA vaccine for *P. aeruginosa* in this study provides a promising framework for future research in the field of vaccination against this bacterium. However, it is important to note that further *in vitro* and *in vivo* studies are necessary to confirm the findings of this study and to evaluate the vaccine’s safety, efficacy and potential limitations in real-world scenarios. Successful development of an effective vaccine against *P. aeruginosa* could have significant implications for public health by reducing the morbidity and mortality associated with this pathogen. The proposed vaccine’s high predicted population coverage and strong affinity for immune receptors TLR-4 and TLR-3 suggest that it may be a promising approach to combat *P. aeruginosa* infections. Overall, this study highlights the potential of *in silico* approaches for vaccine design and provides valuable insights into the development of effective vaccines against *P. aeruginosa*.

**Key Points**

- In designing an effective mRNA vaccine for *P. aeruginosa*, surface proteins critical for binding and entering the bacteria into host cells could serve as suitable targets.
- The results indicated that the designed mRNA construct could be an effective and promising vaccine that requires laboratory and clinical trials.
- The proposed design of a novel multi-epitope mRNA vaccine for *P. aeruginosa* in this study provides a promising framework for future research in the field of vaccination against this bacterium.

## Supplementary Material

Supplementary_file_1_secondary_structure_psipredChart_bbad502

## Data Availability

The data that support the findings of this study are available on request from the corresponding author.

## References

[1] Das S, Howlader DR, Zheng Q, et al. Development of a broadly protective, self-adjuvanting subunit vaccine to prevent infections by Pseudomonas aeruginosa. Front Immunol 2020;11: 583008. 33281815 10.3389/fimmu.2020.583008 PMC7705240

[2] Rad ZR, Rad ZR, Goudarzi H, et al. Detection of New Delhi metallo-β-lactamase-1 among Pseudomonas aeruginosa isolated from adult and pediatric patients in Iranian hospitals. Gene Reports 2021;23:101152.

[3] Anantharajah A, Mingeot-Leclercq M-P, Van Bambeke F. Targeting the type three secretion system in Pseudomonas aeruginosa. Trends Pharmacol Sci 2016;37(9):734–49. 27344210 10.1016/j.tips.2016.05.011

[4] Hart RJ, Morici LA. Vaccination to prevent Pseudomonas aeruginosa bloodstream infections. Front Microbiol 2022;13:870104. 35418967 10.3389/fmicb.2022.870104 PMC8996235

[5] Huang W, Hamouche JE, Wang G, et al. Integrated genome-wide analysis of an isogenic pair of Pseudomonas aeruginosa clinical isolates with differential antimicrobial resistance to ceftolozane/tazobactam, ceftazidime/avibactam, and piperacillin/tazobactam. Int J Mol Sci 2020;21(3):1026. 32033143 10.3390/ijms21031026 PMC7037351

[6] Golmoradi Zadeh R, Mirshekar M, Sadeghi Kalani B, et al. The expression of type II TA system genes following persister cell formation in Pseudomonas aeruginosa isolates in the exponential and stationary phases. Arch Microbiol 2022;204(8):451. 35781545 10.1007/s00203-022-03038-x

[7] Zadeh RG, Kalani BS, Ari MM, et al. Isolation of persister cells within the biofilm and relative gene expression analysis of type II toxin/antitoxin system in Pseudomonas aeruginosa isolates in exponential and stationary phases. J Glob Antimicrob Resist 2022;28:30–7. 34922056 10.1016/j.jgar.2021.11.009

[8] Cabral MP, Correia A, Vilanova M, et al. A live auxotrophic vaccine confers mucosal immunity and protection against lethal pneumonia caused by Pseudomonas aeruginosa. PLoS Pathog 2020;16(2):e1008311. 32040500 10.1371/journal.ppat.1008311 PMC7034913

[9] Killough M, Rodgers AM, Ingram RJ. Pseudomonas aeruginosa: recent advances in vaccine development. Vaccine 2022; 10(7):1100. 10.3390/vaccines10071100 PMC9320790 35891262

[10] Gonzaga ZJC, Merakou C, DiGiandomenico A, et al. A Pseudomonas aeruginosa-derived particulate vaccine protects against P. Aeruginosa infection. Vaccine 2021;9(7):803. 10.3390/vaccines9070803 PMC8309987 34358220

[11] Tandrup Schmidt S, Foged C, Smith Korsholm K, et al. Liposome-based adjuvants for subunit vaccines: formulation strategies for subunit antigens and immunostimulators. Pharmaceutics 2016;8(1):7. 26978390 10.3390/pharmaceutics8010007 PMC4810083

[12] Mohammadzadeh R, Shivaee A, Ohadi E, Kalani BS. In silico insight into the dominant type II toxin–antitoxin systems and Clp proteases in listeria monocytogenes and designation of derived peptides as a novel approach to interfere with this system. Int J Pept Res Ther. 2020;26:613–23.

[13] Asadollahi P, Sadeghifard N, Kazemian H, et al. In silico study of the proteins involved in the persistence of Brucella spp. Curr Drug Discov Technol 2023;20(1):1–13. 35929636 10.2174/1570163819666220805161821

[14] Asadollahi P, Pakzad I, Ghafourian S, et al. In silico investigation of Lon protease as a promising therapeutic target. Drug Res 2022;72(04):180–8. 10.1055/a-1713-3137 35042266

[15] Al TH . Novel in silico mRNA vaccine design exploiting proteins of M. tuberculosis that modulates host immune responses by inducing epigenetic modifications. Sci Rep 2022;12(1):4645. 35301360 10.1038/s41598-022-08506-4 PMC8929471

[16] Asadollahi P, Pakzad I, Sadeghifard N, et al. Immunoinformatics insights into the internalin A and B proteins to design a multi-epitope subunit vaccine for L. monocytogenes. Int J Pept Res Ther 2022;28(1):47.

[17] Chaudhary N, Weissman D, Whitehead KA. mRNA vaccines for infectious diseases: principles, delivery and clinical translation. Nat Rev Drug Discov 2021;20(11):817–38. 34433919 10.1038/s41573-021-00283-5 PMC8386155

[18] Dey J, Mahapatra SR, Patnaik S, et al. Molecular characterization and designing of a novel multiepitope vaccine construct against Pseudomonas aeruginosa. Int J Pept Res Ther 2022;28(2):49. 35069055 10.1007/s10989-021-10356-z PMC8762192

[19] Fakoor MH, Mousavi Gargari SL, Owlia P, Sabokbar A. Protective efficacy of the OprF/OprI/PcrV recombinant chimeric protein against Pseudomonas aeruginosa in the burned BALB/c mouse model. Infect Drug Resist 2020;13:1651–61. 32606816 10.2147/IDR.S244081 PMC7294051

[20] Faezi S, Bahrmand AR, Mahdavi M, et al. Development of a novel anti-adhesive vaccine against Pseudomonas aeruginosa targeting the C-terminal disulfide loop of the pilin protein. Int J Mol Cell Med 2017;6(2):96–108. 28890886 10.22088/acadpub.BUMS.6.2.4 PMC5581551

[21] Faezi S, Nikokar I, Elmi A, et al. Molecular characterization and functional analysis of the PilQ380-706: a novel secretin domain in Pseudomonas aeruginosa. Avicenna J Med Biotechnol 2018;10(1):34–40. 29296265 PMC5742652

[22] Jurado-Martín I, Sainz-Mejías M, McClean S. Pseudomonas aeruginosa: an audacious pathogen with an adaptable arsenal of virulence factors. Int J Mol Sci 2021;22(6):3128. 33803907 10.3390/ijms22063128 PMC8003266

[23] Song WS, Yoon S-i. Crystal structure of FliC flagellin from Pseudomonas aeruginosa and its implication in TLR5 binding and formation of the flagellar filament. Biochem Biophys Res Commun 2014;444(2):109–15. 24434155 10.1016/j.bbrc.2014.01.008

[24] Naveed M, Jabeen K, Naz R, et al. Regulation of host immune response against Enterobacter cloacae proteins via computational mRNA vaccine design through transcriptional modification. Microorganisms 2022;10(8):1621. 36014038 10.3390/microorganisms10081621 PMC9415879

[25] Naveed M, Hassan J-u, Ahmad M, et al. Designing mRNA-and peptide-based vaccine construct against emerging multidrug-resistant Citrobacter freundii: a computational-based subtractive proteomics approach. Medicina 2022;58(10):1356. 36295517 10.3390/medicina58101356 PMC9610710

[26] Wood SJ, Kuzel TM, Shafikhani SH. Pseudomonas aeruginosa: infections, animal modeling, and therapeutics. Cell 2023;12(1):199. 10.3390/cells12010199 PMC9818774 36611992

[27] Sainz-Mejías M, Jurado-Martín I, McClean S. Understanding Pseudomonas aeruginosa–host interactions: the ongoing quest for an efficacious vaccine. Cell 2020;9(12):2617. 10.3390/cells9122617 PMC7762141 33291484

[28] Worgall S . 40 years on: have we finally got a vaccine for Pseudomonas aeruginosa? Future Microbiol 2012;7(12):1333–5. 23231481 10.2217/fmb.12.106

[29] Sharma A, Krause A, Worgall S. Recent developments for pseudomonas vaccines. Hum Vaccin 2011;7(10):999–1011. 21941090 10.4161/hv.7.10.16369 PMC3360073

[30] Shamsinejad FS, Zafari Z. Prediction of potential drug targets and vaccine candidates against antibiotic-resistant Pseudomonas aeruginosa. Int J Pept Res Ther 2022;28(6):160. 36406282 10.1007/s10989-022-10463-5 PMC9640888

[31] Shahrear S, Islam ABMMK. Immunoinformatics guided modeling of CCHF_GN728, an mRNA-based universal vaccine against Crimean-Congo hemorrhagic fever virus. Comput Biol Med 2022;140:105098. 34875407 10.1016/j.compbiomed.2021.105098

[32] Pardi N, Hogan M, Porter F, Weissman D. mRNA vaccines—a new era in vaccinology. NatRev Drug Discov 2017;17:261–79. 10.1038/nrd.2017.243 PMC5906799 29326426

[33] Shahrear S, Islam ABMMK. Modeling of MT. P495, an mRNA-based vaccine against the phosphate-binding protein PstS1 of Mycobacterium tuberculosis. Mol Divers 2022;27:1613–32. 36006502 10.1007/s11030-022-10515-4 PMC9406248

