# Supplementary figures and images for: Development of innovative multi-epitope mRNA vaccine against Pseudomonas aeruginosa using in silico approaches
Source: Brief Bioinform. 2024 Jan 6;25(1):bbad502. doi: 10.1093/bib/bbad502 (PMC10772946; doi:10.1093/bib/bbad502)

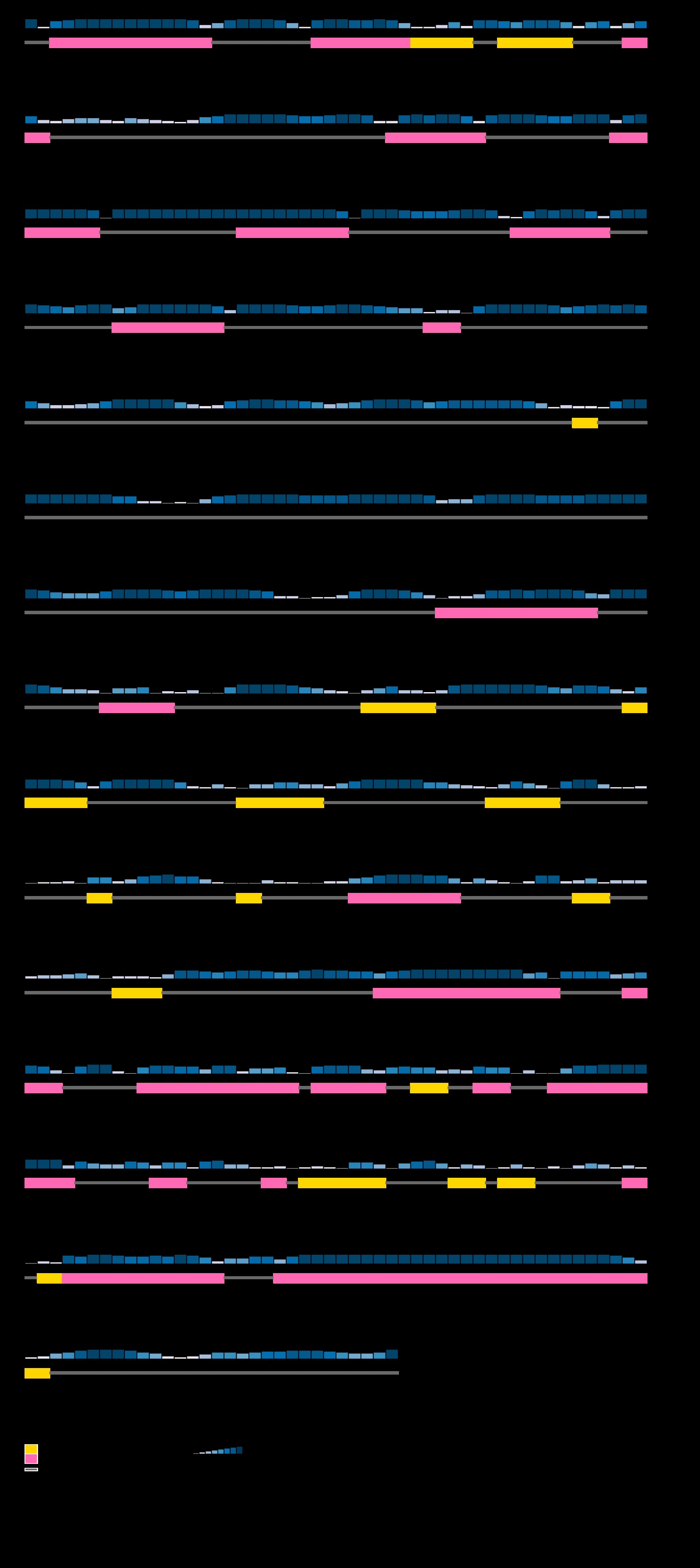

Supplement: Supplementary_file_1_secondary_structure_psipredChart_bbad502 [file supplementary_file_1_secondary_structure_psipredchart_bbad502.jpeg]
